# Temperature: a driving factor for *Meloidogyne floridensis* migration toward different hosts

**DOI:** 10.21307/jofnem-2021-074

**Published:** 2021-08-12

**Authors:** Diego A. H. S. Leitão, Elvira M. R. Pedrosa, Donald W. Dickson, Ana Karina S. Oliveira, Mario Monteiro Rolim

**Affiliations:** 1 Entomology and Nematology Department, University of Florida, Gainesville, FL, 32608; 2 Agricultural Engineering Department, Federal Rural University of Pernambuco, Recife, Pernambuco, 52171-900, Brazil

**Keywords:** Behavior, Mobility, Peach root-knot nematode, Soil column *Tagete patula*

## Abstract

The peach root-knot nematode, *Meloidogyne floridensis*, is an emerging species and may become a threat to peach growers if contamination and spread are not avoided. The influence of temperature and two plants – tomato (*Solanum lycopersicum*) and French marigold (*Tagete patula*) – on the vertical migration of second-stage juveniles (J2) of *M. floridensis* was studied using 14-cm long segmented soil columns. Plants were transplanted into cups attached to the top of each column. Nylon meshes were placed between cups and columns to prevent downward root growth. About 1,000 freshly hatched J2 were injected into the base of the columns and then the columns were transferred to growth chambers at 20 and 26°C under a completely randomized block design with four replicates. The number of J2 in each ring of the columns as well as inside tomato or marigold roots was recorded at 3, 6, 9, and 12 days after injection (DAI). Nematode data were subjected to a repeated measures MANOVA. The presence of plants did not improve J2 migration as compared to control. *M. floridensis* migration was best at 20°C at first, with J2 migrating more than 13 cm as soon as 3 DAI, while it took 9 DAI for J2 to migrate long distances at 26°C. The distribution of J2 along the columns was similar at both temperatures at 12 DAI. Temperature had no influence on J2 penetration. French marigold did not hinder J2 migration, but fewer J2 penetrated its roots.

Plant-parasitic nematodes are microorganisms associated with economically important agricultural crops. Their parasitism may cause considerable yield suppressions, 9 to 15% of worldwide production (Nicol et al., 2011), thus representing a major constraint for global food security. Root-knot nematodes (*Meloidogyne* spp.) have been ranked first among genera of plant-parasitic nematodes that threatens world agriculture (Jones et al., 2013).

A new emerging species, *Meloidogyne floridensis*, has been described by Handoo et al. (2004), parasitizing *M. incognita*- and *M. javanica*-resistant peach rootstocks in Florida (Nyczepir and Thomas, 2009; Smith et al., 2015). The nematode was given the common name peach root-knot nematode. Further studies have shown that several other horticultural crops, e.g. tomato (*Solanum lycopersicum*) (Brito et al., 2015; Stanley et al., 2009), are hosts for this root-knot nematode species, while marigold (*Tagetes* spp.) was reported as a nonhost (Kokalis-Burelle and Nyczepir, 2004). The peach root-knot nematode was thought to be restricted to Florida, but very recently it has been reported in almond orchards in California (Westphal et al., 2019) and peach orchards in South Carolina (Reighard et al., 2019).

Before entering the roots, the J2 migrate through the porous space of the soil matrix and are subjected to its environmental conditions, some of which are reported to influence J2 migration and survival (Prot, 1980; Wallace, 1968). Temperature is one of the main factors that affect plant-parasitic nematodes throughout their developmental cycle (Karssen et al., 2013). Migration studies under temperature gradients demonstrated that *M. incognita* moved toward warmer areas (Robinson, 1994). Prot and van Gundy (1981) reported that *M. incognita* migration reached a maximum at 20°C, whereas *M. hapla* migrated well at lower temperatures, and *M. javanica* migration was increased at 25°C (Wallace, 1966). These results imply that there is an optimal temperature range for migration, and it might be species-specific (Robinson and Perry, 2006).

Soil column assays allow for determining the ability of J2 to migrate toward host roots overtime under controlled conditions, which enhanced the realism of laboratory results to those under field conditions (Spence et al., 2008). In general, the vertical migration is determined by the number of J2 that move from the injection point to different distances along the column over a given time interval. Prot (1976) reported that J2 of *M. javanica* were able to migrate 50 cm, both horizontal and vertically, and parasitize tomato plants. Dalzell et al. (2011) reported that *M. incognita* showed a preferential migratory pattern toward tomato when compared to treatments with no plants. However, no preferential migration was observed for *M. chitwoodi* in columns with and without tomato (Pinkerton et al., 1987).

Although experimental assays have broadened our knowledge on nematode migration, little is known about *M. floridensis* behavior within soil. Therefore, our goal was to determine how temperature (20 and 26°C) and host (tomato) and non-host (French marigold) plants influenced the migration of J2 of peach root-knot nematode over time. Our objectives were to determine whether: (i) high temperature increases the rate of *M. floridensis* migration, (ii) migration patterns will be different under each plant, with J2 being repelled by marigold, and (iii) J2 migrate long distances even without the presence of a host plant.

## Materials and methods

### Nematode inoculum

*Meloidogyne floridensis* isolates were maintained on tomato (*Solanum lycopersicum* ‘Cobra’) grown in sandy soil-filled clay pots in greenhouses at the University of Florida, Gainesville, FL. In total, 60-day old plants were selected, and their roots were washed free of debris with tap water and chopped into 2-cm long pieces. A 0.5% NaOCl solution was used for egg extraction (Hussey and Barker, 1973). The egg solution was poured into a modified Baermann funnel at 27°C in 2-ply facial tissue paper (Kleenex, Neenah, WI, USA) (Rodríguez-Kábana and Pope, 1981). The J2 of *M. floridensis* that hatched during the first 24 hr were discarded to avoid including J2 that might have already been hatched in the suspension and to guarantee that the J2 used in the assays were with the same ‘age’. For the migration assays, a mixture of 3, 2, and 1-day-old J2 were collected on a 25-µm sieve daily for 72 hr and the J2 suspension was kept under refrigeration at 4°C.

### Tomato and French marigold plants

Tomato ‘Cobra’ and French marigold ‘Petite’ seeds were sown (one seed per plant cell) into vermiculite on seedling trays (Park Seed Co., Hodges, SC, USA) and kept under greenhouse conditions (28 ± 3°C, 90–95% relative humidity, and 14:10 hr L:D photoperiod). Four-week-old tomato and French marigold plants were used in the migration assays.

### Experimental apparatus

**Figure 1:**
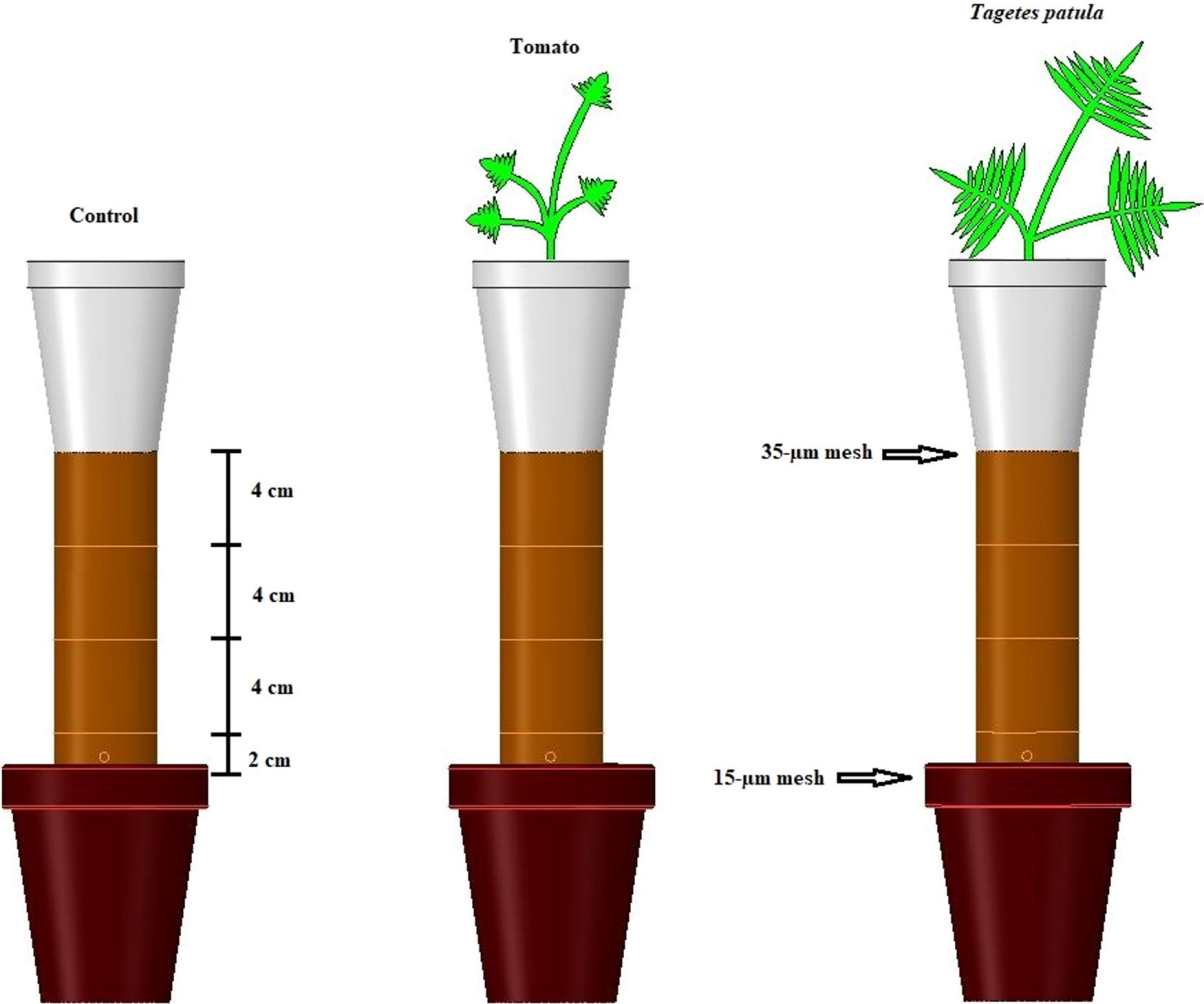
Experimental apparatus used in vertical migration assay of second-stage juveniles (J2) of *Meloidogyne floridensis*. Column consists of three 4.4-cm-d x 4-cm long rings taped together to one 2-cm long ring filled with sandy soil. Four-week-old tomato or *Tagete patula* plants were transplanted to 300-cm^3^ Styrofoam cups and taped to the upper end of the columns – cups with no plants were used as control. Freshly hatched J2 were injected through an injection hole, 1 cm above the base of the column. Arrows indicate where the 35-µm or 15-µm meshes were attached.

The migration of *M. floridensis* was evaluated using polyvinylchloride (PVC) rings taped together with a water-resistant tape (3 M, St. Paul, MN, USA) to assemble segmented columns (Fig. 1). Each apparatus consisted of five compartments (experimental unit): one 2-cm long ring (injection ring), three 4-cm long rings, and a Styrofoam cup on the upper end. A hole was drilled 1 cm above the base of the injection ring to inject J2 into the experimental apparatus. Each column was 14-cm long with 4.4-cm internal diameter and 213-cm^3^ internal volume. Candler sand (96% sand, 2% silt, 2% clay, 0.27% organic matter) collected from a peanut field in Levy County, FL, was heat-pasteurized and then used to fill the columns at 1.2 kg dm^‒3^ soil bulk density and 10% water content by weight. This was considered to simulate field conditions. A 15-µm nylon mesh (Tegape Inc., Curitiba, Brazil) was used to cover the injection ring to avoid juvenile’s movement out of the apparatus. Right after assembling the columns, a parafilm sheet covered the top portion to prevent evaporative water loss until the beginning of the experiment.

Styrofoam cups were used to house the plants. The bottom of the cups was removed and covered with a 35-µm openings nylon mesh to inhibit root growth into the soil columns, whilst allowing J2 to move through the mesh into the soil in the cups (Prot, 1976). Each cup was filled with 300 g of Candler soil at 10% water content and taped to the top of the columns. The root system of each plant was thoroughly rinsed to remove vermiculite debris. Plants were individually transplanted to 300-ml Styrofoam cups which were attached to the top of the columns before J2 injection. Plant-free cups were used as control. The fully assembled columns were placed inside growth chambers and kept at 20 and 26°C with a photoperiod regime of 16 hr light/8 hr dark for 24 hr prior to J2 injection. Temperatures were chosen based on common soil temperatures in Florida (26°C) and observe the migration of *M. floridensis* in 20°C, reported as best for maximum migration of *M. incognita* (Prot and van Gundy, 1981). The growth chamber conditions were maintained throughout the experiment.

### Experimental set-up

Columns were arranged in a completely randomized block design with four replicates. Each replicate was performed in a separate growth chamber. As columns were disassembled 3, 6, 9, and 12 days after injection (DAI), a total of 96 columns and 480 experimental units were used. Freshly hatched J2 were counted under a stereomicroscope Labophot (Nikon, Tokyo, Japan) at 100 × magnification, and the suspension was adjusted to 500 ± 50 J2 ml^**–**1^. An aliquot of 2 ml of J2 suspension (1,000 ± 100 J2) were injected into the columns through the hole in the injection ring after acclimatization of soil columns for 24 hr inside the growth chambers. Following injection, the holes were sealed with water-resistant tape. No water was added during the first 24 hr to prevent unnecessary nematode percolation. Columns were watered daily to maintain the soil water content at 10% by weight; the amount of water added was equal to that lost by evapotranspiration (Pudasaini et al., 2007). To avoid temperature fluctuation inside the columns during and after daily irrigation, a water bottle was kept inside both growth chambers.

When the columns were disassembled, a spatula was used to disconnect and not to mix the soil of each ring. J2 were extracted separately from each ring and the Styrofoam cup by the centrifugal-flotation method (Jenkins, 1964). After each extraction, the nylon meshes (35 and 15 µm) were checked under a stereomicroscope for trapped J2. The numbers of recovered and active J2 in each ring were recorded; any J2 that showed movement regardless of its intensity was classified as active J2. The root systems of tomato and French marigold plants were washed free of debris with tap water and stained with acid fuchsin (Byrd et al., 1983) to determine the number of J2 per gram of fresh roots using SteREO v. 20 stereomicroscope (Carl Zeiss, Göttingen, Germany). Additionally, fresh shoot and root weights were recorded and correlated to nematode migration.

### Statistical analysis

Nematode count data were subjected to transformation (√x + 0.5) before conducting statistical analysis to meet MANOVA assumptions of normality (Shapiro–Wilk test) and homoscedasticity (Levene’s test). The effects of plants (plant-free, tomato, and French marigold), distance migrated (<1, 1–5, 5–9, 9–13, and >13 cm), temperature (20 and 26°C) and time (3, 6, 9, and 12 DAI) on *M. floridensis* vertical migration were analyzed through a repeated measures MANOVA. A chi-square (*χ*^2^) test was performed to compare the distribution of J2 along the columns for significant effects and interactions, while Tukey’s multiple comparison test was used to compare the penetration of J2 into tomato and marigold roots. Additionally, Pearson’s correlation coefficients between plant growth variables and J2 inside roots were also determined. All statistical analyses were performed in RStudio 1.3 environment, without the use of any packages (RStudio Inc, Boston, MA, 2015).

## Results

Less than 8% of all injected J2 (96,000) were recovered throughout the experiment, with an average of 1.86% recovery rate per column. The presence of tomato or French marigold plants did not improve the migration rate of J2 when compared with the control (*p* ≥ 0.05). The distribution of recovered J2 along the columns was significantly influence by temperature and time (*p* < 0.05), while the migration of active J2 differed between temperatures (*p* < 0.05) and rings (*p* < 0.0001, Table 1) over time. No J2 were found trapped in either nylon meshes.

**Table 1.** Repeated measures MANOVA summary of the effects of plants, distance migrated (rings), temperature and time on vertical migration of recovered and active second-stage juveniles (J2) of *Meloidogyne floridensis* along PVC columns filled with sandy soil.

		Recovered J2				Active J2			
Source	df	SS	MS	*F*	*p* > *F*	SS	MS	*F*	*p* > *F*
Block	3	166.05	55.35	20.22	*<0.0001*	167.94	55.98	22.24	*<0.0001*
Temperature (Temp)	1	60.22	60.22	22.00	*<0.0001*	79.11	79.11	31.43	*<0.0001*
Plant	2	6.72	3.36	1.23	0.2941	2.38	1.19	0.47	0.6248
Rings	4	966.36	241.59	88.26	*<0.0001*	696.60	174.15	69.18	*<0.0001*
Temp:Plant	2	1.42	0.71	0.26	0.7713	0.44	0.22	0.09	0.9160
Temp:Rings	4	13.32	3.33	1.22	0.3038	10.88	2.72	1.08	0.3650
Plant:Rings	8	15.04	1.88	0.69	0.7030	11.52	1.44	0.57	0.8012
Temp:Plant:Rings	8	33.20	4.15	1.52	0.1497	32.56	4.07	1.62	0.1181
Time	3	41.46	13.82	5.05	*0.0019*	64.92	21.64	8.60	*<0.0001*
Time:Temp	3	11.82	3.94	1.44	0.2308	28.26	9.42	3.74	*0.0114*
Time:Plant	6	18.66	3.11	1.14	0.3413	31.62	5.27	2.10	0.0532
Time:Rings	12	271.08	22.59	8.25	*<0.0001*	199.32	16.61	6.60	*<0.0001*
Time:Temp:Plant	6	17.70	2.95	1.08	0.3753	9.18	1.53	0.61	0.7235
Time:Temp:Rings	12	64.56	5.38	1.96	*0.0266*	39.36	3.28	1.31	0.2134
Time:Plant:Rings	24	72.00	3.00	1.10	0.3444	50.40	2.10	0.83	0.6935
Time:Temp:Plant:Rings	24	56.40	2.35	0.86	0.6606	62.40	2.60	1.03	0.4217

**Notes:** df: degree of freedom; SS: sum of squares; MS: mean square, *p* > *F*: significance level of *F* test.

**Figure 2:**
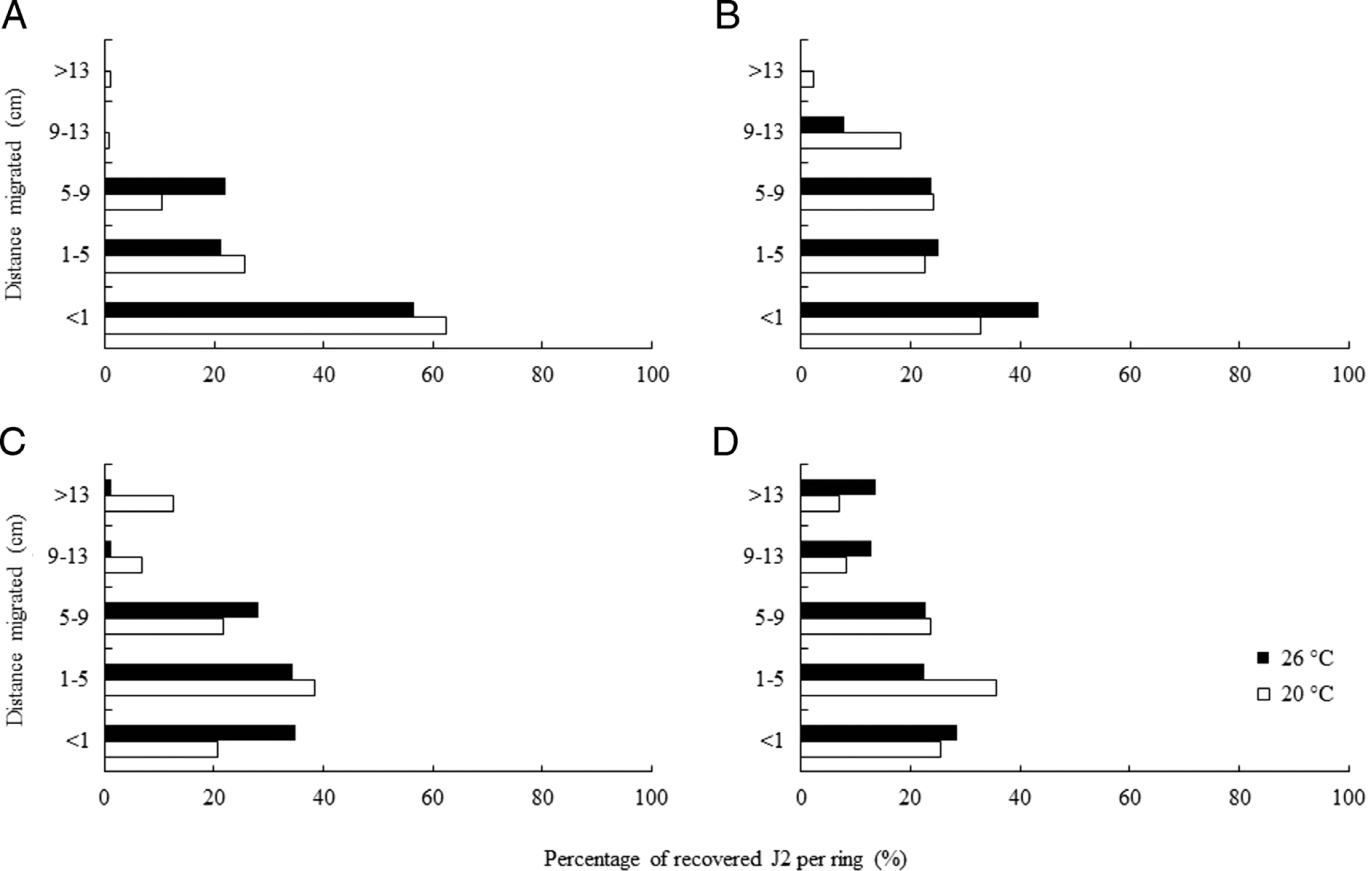
Distribution of recovered second-stage juveniles (J2) of *Meloidogyne floridensis* as a function of distance migrated and temperature at 3 (A), 6 (B), 9 (C), and 12 (D) days after injection. Nematode distribution along the column (4-cm long rings taped together) over time and at 20 and 26°C was statistically different when data were subjected to *Χ*^2^ test (*p* < 0.01).

At 3 DAI, more than 50% (775) of recovered J2 were found at the injection ring (< 1 cm) regardless of temperature; however, none were observed above 9 cm at 26°C, whereas less than 1% (11) migrated > 13 cm at 20°C (Fig. 2A). At 6 DAI, although most J2 still remained at the injection ring, 8% (75) were able to migrate up to 13 cm at 26°C; the percentage of recovered J2 at 20°C decreased at the injection ring and became evenly distributed from 1 to 13 cm (Fig. 2B). There was a significant increase (10%) on the percentage of J2 migrating > 13 cm at 20°C at 9 DAI, whereas J2 concentrated within the first 9 cm of the columns at 26°C (Fig. 2C). By the end of the experiment, recovered J2 were uniformly distributed along the columns, with similar recovery percentages at distances of up to 13 and > 13 cm at both temperatures (Fig. 2D).

**Figure 3:**
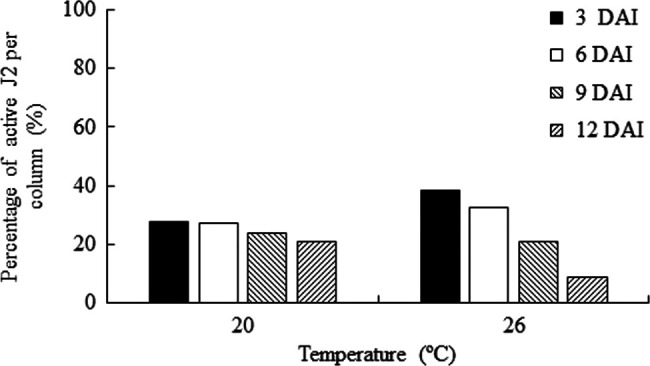
Percentage of active second-stage juveniles (J2) of *Meloidogyne floridensis* recovered at 3, 6, 9, and 12 days after injection (DAI) at 20 and 26°C from sandy soil contained in PVC columns. The columns were comprised of 4-cm long x 4.4-cm internal diameter rings. Nematode distribution over time and at both temperatures was statistically different when data were subjected to *Χ*^2^ test (*p* < 0.01).

At 20°C, the percentage of active J2 decreased from 27% (953) to 21% (726) at 3 and 12 DAI, respectively, whereas there was a 30% (from 732 J2 at 3 DAI to 360 J2 at 12 DAI) reduction on the percentage of active J2 at 26°C. At 3 and 6 DAI greater percentages of J2 were recovered from columns at 26°C, however this behavior changed from 9 DAI onward, with 21% (726) and 8% (360) of active J2 recovered at 20 and 26°C, respectively, at 12 DAI (Fig. 3).

**Figure 4:**
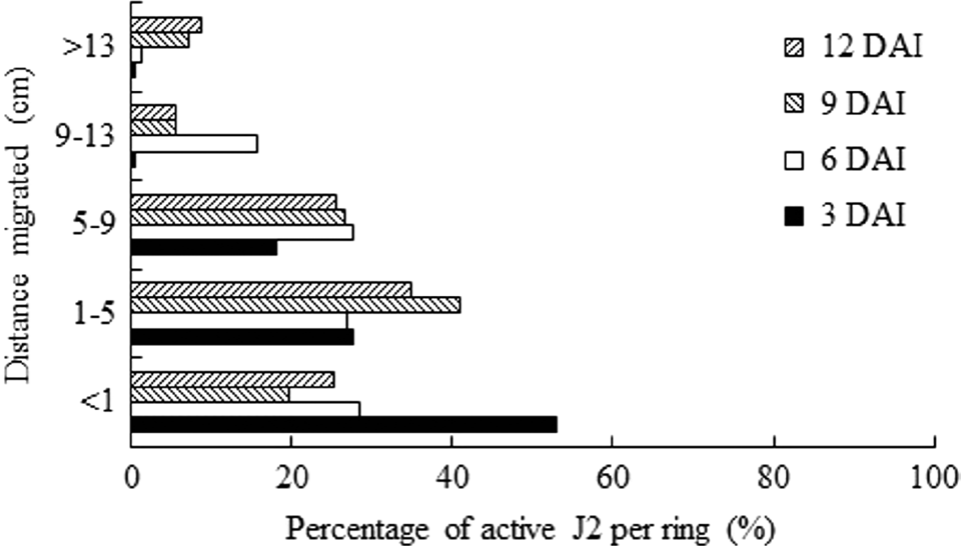
Distribution of active second-stage juveniles (J2) of *Meloidogyne floridensis* as a function of distance migrated at 3, 6, 9, and 12 days after injection (DAI). As there was no significant interaction with temperature, 20 and 26°C data were combined. Nematode distribution along the columns (4-cm long rings taped together) over time was statistically different when data were subjected to *Χ*^2^ test (*p* < 0.01).

There was no significant interaction between temperature, nematode travel distance and time (*p* ≥ 0.05, Table 1), thus data of 20 and 26°C were combined (Fig. 4). The distribution pattern of active J2 along the soil columns changed over time. At 3 DAI, more than 50% (973) active J2 was recovered from the injection ring, while the remaining were concentrated between 1 and 9 cm; only 1% (19) of active J2 were able to migrate more than 9 cm. At 6 DAI, 26 to 28% (465–490) of active J2 were extracted from the first three rings, indicating a progressive and even vertical migration within the first 9 cm; even so, only 1.4% (24) were able to reach the top of the columns (>13 cm). A turning point occurred at 9 DAI, where about 40% (539) of active J2 migrated up to 5 cm and 7% (96) were recovered from the soil inside the Styrofoam cup, which means a migration of more than 13 cm. A similar behavior was observed at 12 DAI, with almost 9% (82) of J2 extracted from distances greater than 13 cm (Fig. 4).

There was no effect of temperature on plant growth variables (data not shown) or penetration of J2 (*p* ≥ 0.05), nor was there a significant interaction between any of the factors (*p* ≥ 0.05, Table 2). Penetration of J2 was greater on tomato than on French marigold plants (*p* < 0.001) with increasing number of J2 inside roots over time (*p* < 0.0001, Table 2).

**Table 2.** Repeated measures MANOVA summary of the effects of temperature and plants on penetration of second-stage juveniles (J2) of *Meloidogyne floridensis* over time.

		J2 inside roots			
Source	df	SS	MS	*F*	*p* > *F*
Block	3	14.60	4.87	5.18	*0.0037*
Temperature (Temp)	1	0.19	0.19	0.20	0.6556
Plant	1	14.62	14.62	15.57	*0.0003*
Temp:Plant	1	2.26	2.26	2.41	0.1279
Time	3	27.89	9.30	9.90	*<0.0001*
Time:Temp	3	3.05	1.02	1.08	0.3663
Time:Plant	3	5.97	1.99	2.12	0.1111
Time:Temp:Plant	3	0.63	0.21	0.22	0.8800

**Notes:** df: degree of freedom; MS: mean square; *p* > *F*: significance level of *F* test; SS: sum of squares.

**Figure 5:**
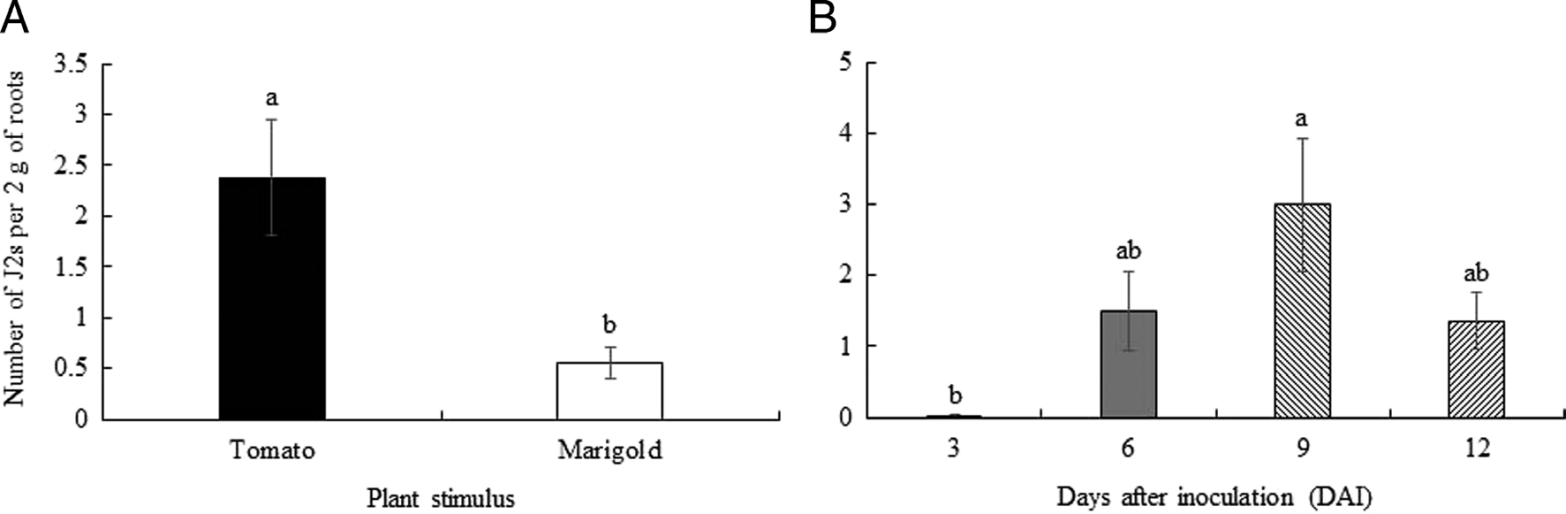
Penetration of second-stage juveniles (J2) of *Meloidogyne floridensis* on tomato or marigold over time after vertically migrating over 13 cm in sandy soil-filled PVC columns. Figures followed by the same letter do not differ according to Tukey’s multiple comparison test (*p* ≥ 0.05) and error bars indicate SEM.

Juveniles were able to parasitize both plant species, however a greater number of J2 were observed inside tomato roots (mean of 2.5 J2 per 2 g of fresh root), whereas less than 1 J2, on average, were counted inside French marigold roots (Fig. 5A). Regarding penetration over time, less than 0.1% of J2 were able to migrate more than 13 cm through the column and parasitize roots regardless of the presence of plants or temperature at 3 DAI. A peak of 3 J2 per 2 g of fresh roots were observed at 9 DAI (Fig. 5B).

## Discussion

The presence of tomato or French marigold plants on the top of the columns did not contribute significantly to the vertical migration of *M. floridensis* J2 compared to plant-free treatments in our experiment. Similarly, the migration rate of *M. chitwoodi* under host-free conditions was found to not differ from that under tomato plants (Pinkerton et al., 1987). However, preferential migration of *M. incognita* and *M. javanica* in soil columns toward suitable hosts was previously reported (Dalzell et al., 2011; Prot, 1976). Feltham and Chaplain (2000) described a migration model for nematodes in restricted domains and pointed out that nematodes may present a biased-random motion strategy, that is, J2 would migrate with a bias toward a host root while simultaneously searching for better food sources. This assumption might explain the results obtained in our experiment.

Although the great majority of J2 were recovered near or at the injection ring, the percentage of J2 at distances greater than 13 cm significantly increased over time, changing from less than 1% at 3 DAI to 9% of active J2 at 12 DAI (Fig. 4). Some studies have reported greater recovery rates of J2 near the plant roots. Prot (1976) observed that 20% of injected J2 of *M. javanica* were able to migrate 50 cm in soil columns with 1.2-cm d rings. Dalzell et al. (2011) reported a recovery rate of nearly 80% of *M. incognita* near tomato rhizosphere in a pipette-bulb assay. On the other hand, Pinkerton et al. (1987) mentioned that less than 0.1% of injected J2 of *M. chitwoodi* and *M. hapla* were able to move 45 cm in soil columns with 8.5-cm d rings. Such different findings might be related to the apparatus dimensions and/or soil physical attributes.

Under natural conditions, the movement of nematodes takes place within an infinite domain (Feltham et al., 2002). However, the evaluation of nematode migratory ability under laboratory conditions occurs in bounded domains and without root growth into the system. Although soil columns have introduced a 3D aspect to migration assays, the dimensional restraint imposed by such columns favors vertical rather than horizontal migration (Spence et al., 2008); therefore, the smaller the internal diameter of the apparatus, the greater the upward migration of J2. According to Hunt et al. (2001), the differences in experimental apparatus and conditions for nematode migration assays make it difficult to create a general model to describe nematode migration within the soil.

While moving within the soil, plant-parasitic nematodes are subjected to environmental changes, including temperature oscillations. In our experiment, J2 migration within soil was best at 20°C for 3, 6, and 9 DAI, where the percentage of recovered J2 was always higher at distances greater than 9 cm when compared to 26°C; by the end of the experiment (12 DAI) J2 distribution was similar between both temperatures (Fig. 1). Several studies have reported that temperature highly influences the migration distance of J2 of plant-parasitic nematodes, and when a gradient is present, they show thermotaxis (Dusenbery, 1988) and tend to migrate toward the warmer end (Robinson, 1994). Prot and van Gundy (1981) have observed maximum vertical migration rates of *M. incognita* at 20°C, while *M. chitwoodi* and *M. hapla* migrated furthest at 18°C (Pinkerton et al., 1987) and *M. javanica* at 25°C (Wallace, 1966). Migratory endoparasitic nematodes have shown similar behavior, i.e. *Pratylenchus penetrans* migration was best at 21°C when compared to 11 and 16°C (Pudasaini et al., 2007).

In our experiment, temperature did not influence the penetration of *M. floridensis* J2 since they are protected from environmental stress when inside healthy host roots (Adam et al., 2014; McSorley, 2003). However, the recovery rate of active J2 from the soil decreased faster at 26°C than at 20°C, suggesting penetration of J2 into tomato or French marigold roots or a greater mortality/inactivity rate of *M. floridensis* J2. In fact, high temperature may hinder root-knot nematode migratory ability (Wallace, 1966). As nematodes are poikilothermic animals (Pudasaini et al., 2007), J2 metabolism increases concomitantly with temperature and their consumption of lipids reserves also increases (Das et al., 2011; Rocha et al., 2016), thus higher inactivity rates might occur.

Tomato is considered a host for several *Meloidogyne* spp. (Seid et al., 2015), including *M. floridensis* (Brito et al., 2015). However, Cetintas et al. (2007) reported that *M. floridensis* J2 showed significant infectivity levels on tomato ‘Florida 47’, whereas they were not as pathogenic on ‘Solar Set’. The authors ruled out low inoculum viability because J2 produced high galling indices on tomato ‘Solar Set’ grown in clay pots. An average of 2.5 J2 of *M. floridensis* per 2 g of fresh roots were found inside tomato ‘Cobra’ in our experiment (Fig. 5), which is comparable to the results observed in microplots experiments using tomato ‘Solar Set’ (Cetintas et al., 2007). Other tomato cultivars have been reported as susceptible (‘Rutgers’ and ‘Talladega’) or resistant (‘Crista’) to *M. floridensis* (Stanley et al., 2009). The rationale behind the discrepancies on host status of different tomato cultivars to *M. floridensis* is still unknown and should be studied to improve peach root-knot nematode management at field scale.

Although we have hypothesized that J2 of *M. floridensis* would be repelled by French marigold exudates, a few J2 were able to penetrate their roots but did not show further development, suggesting French marigold as a nonhost (Kokalis-Burelle and Nyczepir, 2004). The chemotactic responses of active J2 of plant-parasitic nematodes lead them to the vicinity of host root tips or away from non-host plants (Wang et al., 2018). Nematode’s host-finding ability is a complex mechanism that rely on a blend of attractant and repellent compounds exuded by the same plant species (Chaisson and Hallem, 2012; Dutta et al., 2011). Different concentrations of extracts from tomato (Diez and Dusenbery, 1989) and cucumber (Castro et al., 1989) have shown both attractive and repellent properties to *Meloidogyne* spp.

The migratory behavior toward host roots may be species-specific. *Meloidogyne graminicola*, which is a species of root-knot nematode with narrow host range, responded differently to chemical cues from root exudates when compared to species that have a broader host range, such as *M. incognita* (Reynolds et al., 2011). This behavior was also reported for migratory endoparasitic nematodes. The vertical migration of individuals of *P. penetrans* was evaluated under different hosts and column lengths, where a preferential movement under good hosts (maize and bean) was observed regardless of the distance between host and injection point (Pudasaini et al., 2007). Further studies need to focus on understanding how plant-parasitic nematodes perceive exudate compounds as repellent or attractive from a given host (Dutta et al., 2012).

In summary, temperature directly affects the migration of *M. floridensis* J2 within the soil but shows no influence on J2 after they penetrate host tissue. Migration was best at 20°C, but J2 showed similar distribution along the columns over time (i.e. 12 DAI). J2 of *M. floridensis* can migrate great distances (>13 cm) regardless of the presence of plants due to random movement during their host-finding phase. French marigold plants did not repel J2 of *M. floridensis* nor hinder their migration, but fewer J2 were able to penetrate their roots.
